# Supplementary material for: SARS-CoV-2 rebound and post-acute mortality and hospitalization among patients admitted with COVID-19: cohort study
Source: Nat Commun. 2025 Jul 28;16:6924. doi: 10.1038/s41467-025-61737-7 (PMC12304394; doi:10.1038/s41467-025-61737-7)
Supplement: Supplementary file 1 — Supplementary Information [file 41467_2025_61737_MOESM1_ESM.pdf]

Supplementary Table 1. Scores and ICD-9 codes for comorbidities in Charlson Comorbidity Index calculation

Condition	Score	ICD-9 codes
Myocardial infarction	1	410.x, 412.x
Congestive heart failure	1	398.91, 402.01, 402.11, 402.91, 404.01, 404.03, 404.11, 404.13, 404.91, 404.93, 425.4-425.9, 428.x
Peripheral vascular disease	1	093.0, 437.3, 440.x, 441.x, 443.1-443.9, 447.1, 557.1, 557.9, V43.4
Cerebrovascular disease	1	362.34, 430.x-438.x
Dementia	1	290.x, 294.1, 331.2
Chronic pulmonary disease	1	416.8, 416.9, 490.x-505.x, 506.4, 508.1, 508.8
Rheumatic disease	1	446.5, 710.0-710.4, 714.0-714.2, 714.8, 725.x
Peptic ulcer disease	1	531.x-534.x
Mild liver disease	1	070.22, 070.23, 070.32, 070.33, 070.44, 070.54, 070.6, 070.9, 570.x, 571.x, 573.3, 573.4, 573.8, 573.9, V42.7
Diabetes without complication	1	250.0-250.3, 250.8, 250.9
Diabetes with complication	2	250.4-250.7
Hemiplegia or paraplegia	2	334.1, 342.x, 343.x, 344.0-344.6, 344.9
Renal disease	2	403.01, 403.11, 403.91, 404.02, 404.03, 404.12, 404.13, 404.92, 404.93, 582.x, 583.0-583.7, 585.x, 586.x, 588.0, V42.0, V45.1, V56.x
Any malignancy, including lymphoma and leukemia, except malignant neoplasm of skin	2	140.x–172.x, 174.x–195.8, 200.x–208.x, 238.6
Moderate to severe liver disease	3	456.0-456.2, 572.2-572.8
Metastatic solid tumor	6	196.x-199.x
AIDS/HIV	6	042.x-044.x

Supplementary Table 2. ICD-9 codes for ventilatory support

Procedure	ICD-9 codes
Extracorporeal membrane oxygenation (ECMO)	39.65
Other sleep disorder function tests	89.18
Non-invasive mechanical ventilation	93.9
Hyperbaric oxygenation	93.95
Other oxygen enrichment	93.96
Other continuous invasive mechanical ventilation	96.7x
Insertion of endotracheal tube	96.04

Supplementary Table 3. ICD-9 codes for post-acute COVID-19 outcomes

**Post-acute sequelae**	**ICD-9 codes**
Congestive heart failure	398.91, 402.01, 402.11, 402.91, 404.01, 404.03, 404.11, 404.13, 404.91, 404.93, 425.4-425.9, 428.x
Atrial fibrillation	427.3
Coronary artery disease	036.0, 036.1, 410-414, V45.81
Deep vein thrombosis	453
Chronic pulmonary disease	416.8, 416.9, 490.x-505.x, 506.4, 508.1, 508.8
Acute respiratory distress syndrome	518.51-518.53, 518.8x
Interstitial lung disease	135, 495, 515, 516.3, 517.2, 517.8, 714.81
Seizure	333.2, 345, 649.4, 779.0, 780.3
Anxiety	300.0
Post-traumatic stress disorder	308.x-309.x
End-stage renal disease	585.6, 586
Acute kidney injury	584.5-584.9
Pancreatitis	577.0, 577.1

Supplementary Table 4. The principle cause of death of study participants during the observational period.

	Number of patients (n=2897*)
Disease category [3], n (%)	
diseases of the blood and blood-forming organs	21 (0.7)
diseases of the circulatory system	256 (8.8)
diseases of the digestive system	123 (4.2)
diseases of the genitourinary system	132 (4.6)
diseases of the musculoskeletal system and connective tissue	13 (0.4)
diseases of the nervous system and sense organs	7 (0.2)
diseases of the respiratory system	1536 (53.0)
diseases of the skin and subcutaneous tissue	39 (1.3)
endocrine, nutritional and metabolic diseases, and immunity disorders	31 (1.1)
factors influencing health status and contact with health services	8 (0.3)
infectious and parasitic diseases	108 (3.7)
injury and poisoning	42 (1.4)
mental disorders	31 (1.1)
neoplasms	371 (12.8)
symptoms, signs, and ill-defined conditions	179 (6.2)

*The cause of death record of 24 patients is not available in the dataset.

Supplementary Table 5. Additive and multiplicative interaction analysis between nirmatrelvir/ritonavir use and virologic rebound in all study participants for post-acute COVID-19 outcomes occurred 21-365 days after the index date

Outcome	RERI estimate	95% CI	P value	Multiplicative estimate	95% CI	P value
Death	-0.29	(-0.88, 0.29)	0.330	0.68	(0.51, 0.90)	0.006
Composite hospitalization	-0.54	(-1.26, 0.19)	0.146	0.63	(0.43, 0.92)	0.016
Congestive heart failure	-0.41	(-1.88, 1.06)	0.587	0.66	(0.32, 1.36)	0.260
Atrial fibrillation	-1.49	(-2.96, -0.02)	0.046	0.39	(0.19, 0.80)	0.010
Coronary artery disease	-0.20	(-1.35, 0.95)	0.736	0.79	(0.38, 1.64)	0.528
Deep vein thrombosis	NA	NA	NA	NA	NA	NA
Chronic pulmonary disease	0.72	(-0.71, 2.15)	0.322	1.27	(0.51, 3.14)	0.605
Acute respiratory distress syndrome	-0.08	(-1.44, 1.27)	0.906	0.87	(0.41, 1.85)	0.717
Interstitial lung disease	NA	NA	NA	NA	NA	NA
Seizure	-2.17	(-5.40, 1.06)	0.189	0.30	(0.08, 1.14)	0.078
Anxiety	NA	NA	NA	NA	NA	NA
Post-traumatic stress disorder	NA	NA	NA	NA	NA	NA
End-stage renal disease	3.37	(-4.01, 10.75)	0.371	1.27	(0.11, 14.70)	0.846
Acute kidney injury	-0.46	(-1.72, 0.79)	0.468	0.68	(0.31, 1.49)	0.337
Pancreatitis	-8.65	(-23.75, 6.45)	0.262	0.08	(0.01, 1.04)	0.053

RERI: relative excess risk for interaction. In this interaction analysis, the virologic rebound independent variable was coded as 1 for patients with virologic rebound and 0 for patients without virologic rebound. The nirmatrelvir/ritonavir status was coded as 1 for not using nirmatrelvir/ritonavir and 0 for using nirmatrelvir/ritonavir. This coding was to ensure that the two independent variables represented risk factors instead of preventive factors, because preventive factors are not appropriate for the calculation of additive interaction unless recoded to risk factors [1]. The product term of the two independent variables was included in the Cox models. Relative excess risk for interaction (RERI) was calculated to evaluate the additive interaction between virologic rebound and nirmatrelvir/ritonavir [2]. The exponential of the coefficient of the product term was obtained as the measurement of the multiplicative interaction. Statistical analysis with two-sided Wald test.

Supplementary Table 6. Additive and multiplicative interaction analysis between molnupiravir use and virologic rebound in all study participants for post-acute COVID-19 outcomes occurred 21-365 days after the index date

Outcome	RERI estimate	95% CI	P value	Multiplicative estimate	95% CI	P value
Death	0.20	(-0.21, 0.61)	0.339	1.01	(0.78, 1.30)	0.951
Composite hospitalization	0.08	(-0.40, 0.56)	0.738	1.01	(0.70, 1.46)	0.954
Congestive heart failure	0.21	(-0.59, 1.01)	0.613	1.17	(0.61, 2.24)	0.637
Atrial fibrillation	-0.36	(-1.45, 0.73)	0.519	0.73	(0.35, 1.53)	0.410
Coronary artery disease	0.07	(-0.75, 0.89)	0.867	1.02	(0.53, 1.96)	0.942
Deep vein thrombosis	NA	NA	NA	NA	NA	NA
Chronic pulmonary disease	1.15	(-0.06, 2.36)	0.062	2.00	(0.81, 4.94)	0.135
Acute respiratory distress syndrome	0.15	(-1.02, 1.31)	0.807	0.89	(0.47, 1.68)	0.724
Interstitial lung disease	-2.62	(-15.09, 9.85)	0.680	0.25	(0.02, 3.52)	0.306
Seizure	-0.25	(-1.38, 0.89)	0.671	0.77	(0.22, 2.62)	0.672
Anxiety	NA	NA	NA	NA	NA	NA
Post-traumatic stress disorder	0.31	(-1.55, 2.18)	0.741	1.40	(0.17, 11.65)	0.753
End-stage renal disease	1.49	(-0.35, 3.34)	0.113	3.37	(0.60, 18.88)	0.167
Acute kidney injury	-0.16	(-1.07, 0.76)	0.734	0.86	(0.42, 1.76)	0.686
Pancreatitis	-2.68	(-8.01, 2.66)	0.325	0.20	(0.02, 2.48)	0.211

RERI: relative excess risk for interaction. In this interaction analysis, the virologic rebound independent variable was coded as 1 for patients with virologic rebound and 0 for patients without virologic rebound. The molnupiravir status was coded as 1 for not using molnupiravir and 0 for using molnupiravir. This coding was to ensure that the two independent variables represented risk factors instead of preventive factors, because preventive factors are not appropriate for the calculation of additive interaction unless recoded to risk factors [1]. The product term of the two independent variables was included in the Cox models. Relative excess risk for interaction (RERI) was calculated to evaluate the additive interaction between virologic rebound and molnupiravir [2]. The exponential of the coefficient of the product term was obtained as the measurement of the multiplicative interaction. Statistical analysis with two-sided Wald test.

Supplementary Table 7. Additive and multiplicative interaction analysis between nirmatrelvir/ritonavir use and virologic rebound in all study participants for post-acute COVID-19 outcomes occurred 21-365 days after the index date, using alternative definition of virologic rebound (i): a decrease in Ct value of at least 3 units after the end of oral antiviral treatment or treatment completion proxy within 21 days post the index date of virologic rebound.

**Outcome**	**RERI estimate**	**95% CI**	**P value**	**Multiplicative estimate**	**95% CI**	**P value**
Death	-0.54	(-0.98, -0.09)	0.019	0.62	(0.50, 0.78)	<0.001
Composite hospitalization	-0.50	(-1.03, 0.03)	0.065	0.67	(0.50, 0.90)	0.007
Congestive heart failure	-0.06	(-0.89, 0.77)	0.891	0.92	(0.49, 1.72)	0.794
Atrial fibrillation	-0.98	(-2.09, 0.14)	0.086	0.51	(0.28, 0.90)	0.022
Coronary artery disease	-0.33	(-1.41, 0.74)	0.544	0.68	(0.40, 1.17)	0.163
Deep vein thrombosis	0.21	(-1.65, 2.06)	0.826	1.37	(0.23, 8.05)	0.729
Chronic pulmonary disease	-0.33	(-1.50, 0.85)	0.587	0.74	(0.37, 1.49)	0.399
Acute respiratory distress syndrome	-0.40	(-1.43, 0.63)	0.445	0.79	(0.44, 1.40)	0.411
Interstitial lung disease	NA	NA	NA	NA	NA	NA
Seizure	0.90	(-0.89, 2.70)	0.324	1.58	(0.43, 5.75)	0.487
Anxiety	NA	NA	NA	NA	NA	NA
Post-traumatic stress disorder	NA	NA	NA	NA	NA	NA
End-stage renal disease	-2.70	(-10.95, 5.56)	0.522	0.33	(0.05, 2.19)	0.253
Acute kidney injury	-0.44	(-1.41, 0.52)	0.365	0.68	(0.38, 1.22)	0.195
Pancreatitis	-0.74	(-3.98, 2.51)	0.657	0.56	(0.06, 5.42)	0.614

RERI: relative excess risk for interaction. In this interaction analysis, the virologic rebound independent variable was coded as 1 for patients with virologic rebound and 0 for patients without virologic rebound. The nirmatrelvir/ritonavir status was coded as 1 for not using nirmatrelvir/ritonavir and 0 for using nirmatrelvir/ritonavir. This coding was to ensure that the two independent variables represented risk factors instead of preventive factors, because preventive factors are not appropriate for the calculation of additive interaction unless recoded to risk factors [1]. The product term of the two independent variables was included in the Cox models. Relative excess risk for interaction (RERI) was calculated to evaluate the additive interaction between virologic rebound and nirmatrelvir/ritonavir [2]. The exponential of the coefficient of the product term was obtained as the measurement of the multiplicative interaction. Statistical analysis with two-sided Wald test.

Supplementary Table 8. Additive and multiplicative interaction analysis between molnupiravir use and virologic rebound in all study participants for post-acute COVID-19 outcomes occurred 21-365 days after the index date, using alternative definition of virologic rebound (i): a decrease in Ct value of at least 3 units after the end of oral antiviral treatment or treatment completion proxy within 21 days post the index date of virologic rebound.

RERI: relative excess risk for interaction. In this interaction analysis, the virologic rebound independent variable

**Outcome**	**RERI estimate**	**95% CI**	**P value**	**Multiplicative estimate**	**95% CI**	**P value**
Death	-0.09	(-0.39, 0.21)	0.551	0.87	(0.71, 1.06)	0.173
Composite hospitalization	-0.03	(-0.37, 0.32)	0.878	0.95	(0.72, 1.26)	0.743
Congestive heart failure	0.03	(-0.47, 0.53)	0.906	1.05	(0.63, 1.74)	0.858
Atrial fibrillation	-0.30	(-1.04, 0.43)	0.415	0.77	(0.45, 1.31)	0.332
Coronary artery disease	0.13	(-0.51, 0.76)	0.698	1.05	(0.65, 1.70)	0.830
Deep vein thrombosis	-1.10	(-3.37, 1.18)	0.345	0.47	(0.13, 1.66)	0.239
Chronic pulmonary disease	0.38	(-0.58, 1.35)	0.435	1.28	(0.64, 2.56)	0.483
Acute respiratory distress syndrome	-0.39	(-1.35, 0.56)	0.418	0.73	(0.45, 1.21)	0.227
Interstitial lung disease	-0.59	(-13.40, 12.21)	0.928	0.28	(0.02, 3.22)	0.304
Seizure	0.66	(-0.17, 1.49)	0.122	2.13	(0.79, 5.74)	0.136
Anxiety	1.00	(-1.12, 3.12)	0.355	3.37	(0.19, 58.41)	0.403
Post-traumatic stress disorder	1.19	(-0.79, 3.17)	0.239	2.88	(0.53, 15.59)	0.219
End-stage renal disease	0.62	(-0.38, 1.63)	0.225	3.44	(0.63, 18.71)	0.153
Acute kidney injury	-0.15	(-0.88, 0.58)	0.685	0.85	(0.50, 1.44)	0.547
Pancreatitis	-4.63	(-11.82, 2.56)	0.207	0.16	(0.02, 1.09)	0.062

was coded as 1 for patients with virologic rebound and 0 for patients without virologic rebound. The molnupiravir status was coded as 1 for not using molnupiravir and 0 for using molnupiravir. This coding was to ensure that the two independent variables represented risk factors instead of preventive factors, because preventive factors are not appropriate for the calculation of additive interaction unless recoded to risk factors [1]. The product term of the two independent variables was included in the Cox models. Relative excess risk for interaction (RERI) was calculated to evaluate the additive interaction between virologic rebound and molnupiravir [2]. The exponential of the coefficient of the product term was obtained as the measurement of the multiplicative interaction. Statistical analysis with two-sided Wald test.

Aged < 65 years

Outcome	Without VR Events/Number at risk	With VR Events/Number at risk	Risk difference (95% CI)	HR (95% CI)	P value
Death	197/2282	45/352	4.15% (0.48, 7.82)	1.73 (1.15 to 2.62)	0.009
Composite hospitalization	167/1896	30/277	2.02% (−1.85, 5.9)	0.66 (0.34 to 1.26)	0.204
Congestive heart failure	21/2222	3/333	−0.04% (−1.14, 1.05)	0.91 (0.23 to 3.65)	0.892
Atrial fibrillation	16/2253	3/338	0.18% (−0.88, 1.24)	0.52 (0.09 to 2.84)	0.447
Coronary artery disease	32/2208	6/327	0.39% (−1.15, 1.92)	1.04 (0.35 to 3.09)	0.941
Deep vein thrombosis	12/2267	2/350	0.04% (−0.8, 0.89)	2.44 (0.41 to 14.48)	0.326
Chronic pulmonary disease	18/2233	2/345	−0.23% (−1.11, 0.66)	1.14 (0.23 to 5.64)	0.877
Acute respiratory distress syndrome	62/2226	18/339	2.52% (0.04, 5.01)	1.33 (0.60 to 2.98)	0.480
Interstitial lung disease	1/2275	0/352			
Seizure	32/2180	4/339	−0.29% (−1.54, 0.97)	0.95 (0.31 to 2.88)	0.926
Anxiety	2/2276	2/348	0.49% (−0.32, 1.29)	5.65 (0.75 to 42.85)	0.094
Post−traumatic stress disorder	12/2249	0/347			
End−stage renal disease	15/2264	2/348	−0.09% (−0.95, 0.77)	0.39 (0.06 to 2.65)	0.337
Acute kidney injury	34/2239	9/343	1.11% (−0.66, 2.87)	0.84 (0.26 to 2.70)	0.763
Pancreatitis	3/2273	1/351	0.15% (−0.42, 0.73)	1.78 (0.13 to 23.72)	0.661

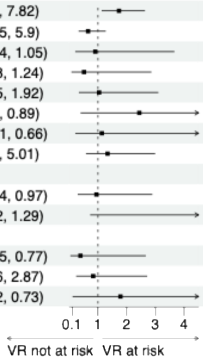

Age >= 65 years

Outcome	Without VR Events/Number at risk	With VR Events/Number at risk	Risk difference (95% CI)	HR (95% CI)	P value
Death	2293/10040	386/1221	8.77% (6.04, 11.51)	1.60 (1.42 to 1.81)	<0.001
Composite hospitalization	1272/7433	183/825	5.07% (2.11, 8.03)	1.32 (1.11 to 1.57)	0.001
Congestive heart failure	385/9326	59/1092	1.27% (−0.13, 2.68)	1.51 (1.12 to 2.03)	0.007
Atrial fibrillation	346/9272	55/1110	1.22% (−0.11, 2.56)	1.47 (1.07 to 2.00)	0.016
Coronary artery disease	383/9282	57/1089	1.11% (−0.28, 2.49)	1.29 (0.95 to 1.75)	0.107
Deep vein thrombosis	64/9973	2/1210	−0.48% (−0.75, −0.2)	0.41 (0.10 to 1.66)	0.209
Chronic pulmonary disease	222/9374	38/1138	0.97% (−0.12, 2.06)	1.30 (0.88 to 1.92)	0.181
Acute respiratory distress syndrome	366/9777	53/1167	0.8% (−0.45, 2.05)	1.07 (0.78 to 1.47)	0.690
Interstitial lung disease	18/10022	4/1218	0.15% (−0.18, 0.48)	1.30 (0.39 to 4.33)	0.668
Seizure	81/9900	16/1189	0.53% (−0.15, 1.21)	1.68 (0.93 to 3.02)	0.084
Anxiety	11/10001	1/1216	−0.03% (−0.2, 0.15)	0.65 (0.08 to 5.41)	0.688
Post−traumatic stress disorder	20/9992	6/1210	0.30% (−0.16, 0.58)	0.92 (0.28 to 3.04)	0.897
End−stage renal disease	43/9981	9/1215	0.31% (−0.19, 0.81)	1.56 (0.71 to 3.41)	0.265
Acute kidney injury	323/9751	44/1175	0.43% (−0.71, 1.57)	1.30 (0.92 to 1.83)	0.137
Pancreatitis	22/10006	7/1215	0.36% (−0.08, 0.79)	2.72 (1.00 to 7.34)	0.049

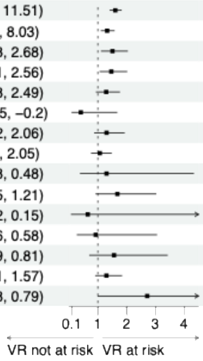

Supplementary Figure 1. Subgroup analysis of the association between virologic rebound and each post-acute COVID-19 outcome in all study participants by age groups for post-acute COVID-19 outcomes 21-365 days after the index date. Cohort with virologic rebound aged < 65 years (n = 352) and cohort without virologic rebound aged < 65 years (n = 2282). Cohort with virologic rebound aged >= 65 years (n = 1221) and cohort without virologic rebound aged >= 65 years (n =10040). Adjusted HRs (square dots) and 95% (error bars) CIs are presented. The dashed vertical line represents the HR of 1.00. Statistical analysis with two-sided Wald test. VR: virologic rebound. CI: confidence interval.

CCI < 4

Outcome	Without VR Events/Number at risk	With VR Events/Number at risk	Risk difference (95% CI)	HR (95% CI)	P value
Death	2161/11465	350/1417	5.85% (3.49, 8.21)	1.56 (1.37 to 1.77)	<0.001
Composite hospitalization	1381/8988	207/1044	4.46% (1.93, 6.99)	1.29 (1.09 to 1.52)	0.002
Congestive heart failure	396/10890	61/1313	1.01% (−0.18, 2.2)	1.52 (1.13 to 2.04)	0.005
Atrial fibrillation	346/10798	55/1317	0.97% (−0.16, 2.1)	1.49 (1.08 to 2.04)	0.014
Coronary artery disease	389/10825	58/1313	0.82% (−0.34, 1.99)	1.22 (0.89 to 1.66)	0.211
Deep vein thrombosis	64/11397	4/1405	−0.28% (−0.59, 0.03)	0.81 (0.29 to 2.27)	0.689
Chronic pulmonary disease	228/10856	38/1343	0.73% (−0.2, 1.66)	1.24 (0.84 to 1.84)	0.285
Acute respiratory distress syndrome	399/11235	67/1370	1.34% (0.15, 2.53)	1.17 (0.88 to 1.57)	0.282
Interstitial lung disease	18/11442	5/1414	0.2% (−0.12, 0.51)	1.52 (0.50 to 4.65)	0.461
Seizure	102/11265	15/1377	0.18% (−0.39, 0.76)	1.16 (0.65 to 2.07)	0.627
Anxiety	13/11426	3/1412	0.1% (−0.15, 0.35)	1.50 (0.38 to 5.92)	0.563
Post−traumatic stress disorder	29/11397	4/1405	0.03% (−0.26, 0.32)	0.90 (0.29 to 2.75)	0.854
End−stage renal disease	52/11421	7/1413	0.04% (−0.35, 0.43)	0.97 (0.41 to 2.30)	0.948
Acute kidney injury	325/11212	49/1380	0.65% (−0.37, 1.68)	1.36 (0.96 to 1.91)	0.080
Pancreatitis	24/11428	7/1411	0.29% (−0.09, 0.66)	2.42 (0.90 to 6.47)	0.079

0.1 1 2 3 4

VR not at risk VR at risk

CCI >= 4

Outcome	Without VR Events/Number at risk	With VR Events/Number at risk	Risk difference (95% CI)	HR (95% CI)	P value
Death	329/857	81/156	13.53% (5.04, 22.02)	2.03 (1.53 to 2.71)	<0.001
Composite hospitalization	58/341	6/58	−6.66% (−15.46, 2.13)	0.93 (0.37 to 2.34)	0.880
Congestive heart failure	10/658	1/112	−0.63% (−2.6, 1.35)	0.79 (0.09 to 6.86)	0.832
Atrial fibrillation	16/727	3/131	0.09% (−2.69, 2.86)	0.97 (0.26 to 3.72)	0.970
Coronary artery disease	26/665	5/103	0.94% (−3.46, 5.35)	1.87 (0.65 to 5.41)	0.245
Deep vein thrombosis	12/843	0/155			
Chronic pulmonary disease	12/751	2/140	−0.17% (−2.33, 1.99)	1.49 (0.30 to 7.47)	0.628
Acute respiratory distress syndrome	29/768	4/136	−0.83% (−3.98, 2.31)	0.74 (0.23 to 2.36)	0.614
Interstitial lung disease	1/855	0/156			
Seizure	11/815	5/151	1.96% (−1, 4.92)	3.95 (1.19 to 13.12)	0.025
Anxiety	0/851	0/152			
Post−traumatic stress disorder	3/844	1/152	0.3% (−1.04, 1.65)	0.60 (0.06 to 6.15)	0.668
End−stage renal disease	6/824	4/150	1.94% (−0.7, 4.58)	3.94 (0.87 to 17.81)	0.075
Acute kidney injury	32/778	4/138	−1.21% (−4.34, 1.91)	0.89 (0.29 to 2.73)	0.833
Pancreatitis	1/851	1/155	0.53% (−0.75, 1.81)	4.14 (0.26 to 66.10)	0.315

0.1 1 2 3 4

VR not at risk VR at risk

Supplementary Figure 2. Subgroup analysis of the association between virologic rebound and each post-acute COVID-19 outcome in all study participants by Charlson Comorbidity Index categories for post-acute COVID-19 outcomes 21-365 days after the index date. Cohort with virologic rebound and CCI < 4 (n = 1417) and cohort without virologic rebound and CCI < 4 (n = 11465). Cohort with virologic rebound and CCI >= 4 (n = 156) and cohort without virologic rebound and CCI >= 4 (n = 857). Adjusted HRs (square dots) and 95% (error bars) CIs are presented. Adjusted HRs (square dots) and 95% (error bars) CIs are presented. The dashed vertical line represents the HR of 1.00. Statistical analysis with two-sided Wald test. CCI: Charlson Comorbidity Index. VR: virologic rebound. CI: confidence interval.

Vaccinated patients

Outcome	Without VR Events/Number at risk	With VR Events/Number at risk	Risk difference (95% CI)	HR (95% CI)	P value
Death	1594/9053	305/1186	8.11% (5.5, 10.72)	1.75 (1.51 to 2.02)	<0.001
Composite hospitalization	1000/6986	163/849	4.88% (2.11, 7.66)	1.36 (1.13 to 1.65)	0.001
Congestive heart failure	293/8540	47/1086	0.9% (−0.37, 2.17)	1.52 (1.08 to 2.14)	0.016
Atrial fibrillation	255/8512	46/1095	1.21% (−0.04, 2.45)	1.53 (1.06 to 2.20)	0.023
Coronary artery disease	279/8503	45/1079	0.89% (−0.36, 2.14)	1.23 (0.87 to 1.74)	0.248
Deep vein thrombosis	58/8987	4/1175	−0.3% (−0.68, 0.07)	1.17 (0.41 to 3.31)	0.773
Chronic pulmonary disease	166/8575	28/1124	0.56% (−0.4, 1.51)	1.30 (0.83 to 2.04)	0.258
Acute respiratory distress syndrome	286/8834	52/1135	1.34% (0.07, 2.62)	1.24 (0.89 to 1.73)	0.204
Interstitial lung disease	15/9035	3/1183	0.09% (−0.21, 0.39)	1.58 (0.39 to 6.43)	0.520
Seizure	83/8877	15/1156	0.36% (−0.32, 1.04)	1.39 (0.77 to 2.53)	0.274
Anxiety	11/9020	2/1178	0.05% (−0.2, 0.29)	1.39 (0.27 to 7.08)	0.692
Post−traumatic stress disorder	25/8997	4/1174	0.06% (−0.29, 0.41)	0.73 (0.23 to 2.28)	0.582
End−stage renal disease	35/8994	8/1181	0.29% (−0.2, 0.77)	1.40 (0.53 to 3.69)	0.501
Acute kidney injury	243/8823	37/1147	0.47% (−0.61, 1.55)	1.28 (0.85 to 1.91)	0.233
Pancreatitis	17/9021	7/1181	0.4% (−0.04, 0.85)	4.30 (1.48 to 12.51)	0.007

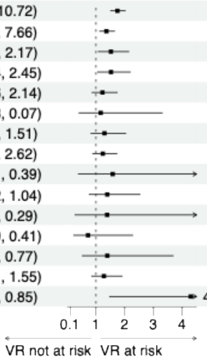

Unvaccinated patients

Outcome	Without VR Events/Number at risk	With VR Events/Number at risk	Risk difference (95% CI)	HR (95% CI)	P value
Death	896/3269	126/387	5.15% (0.24, 10.06)	1.35 (1.10 to 1.66)	0.004
Composite hospitalization	439/2343	50/253	1.03% (−4.13, 6.18)	0.99 (0.71 to 1.37)	0.938
Congestive heart failure	113/3008	15/339	0.67% (−1.62, 2.96)	1.39 (0.79 to 2.45)	0.252
Atrial fibrillation	107/3013	12/353	−0.15% (−2.15, 1.85)	1.05 (0.56 to 1.98)	0.881
Coronary artery disease	136/2987	18/337	0.79% (−1.73, 3.3)	1.16 (0.65 to 2.06)	0.614
Deep vein thrombosis	18/3253	0/385			
Chronic pulmonary disease	74/3032	12/359	0.9% (−1.04, 2.84)	1.27 (0.64 to 2.52)	0.499
Acute respiratory distress syndrome	142/3169	19/371	0.64% (−1.72, 3)	0.93 (0.55 to 1.57)	0.784
Interstitial lung disease	4/3262	2/387	0.39% (−0.33, 1.12)	1.87 (0.30 to 11.67)	0.505
Seizure	30/3203	5/372	0.41% (−0.81, 1.62)	1.46 (0.54 to 3.94)	0.452
Anxiety	2/3257	1/386	0.2% (−0.32, 0.71)	1.87 (0.15 to 23.65)	0.630
Post−traumatic stress disorder	7/3244	1/383	0.05% (−0.49, 0.58)	1.34 (0.14 to 12.58)	0.797
End−stage renal disease	23/3251	3/382	0.08% (−0.85, 1.01)	1.12 (0.32 to 3.96)	0.860
Acute kidney injury	114/3167	16/371	0.71% (−1.45, 2.88)	1.29 (0.74 to 2.24)	0.373
Pancreatitis	8/3258	1/385	0.01% (−0.52, 0.55)	0.73 (0.09 to 6.28)	0.776

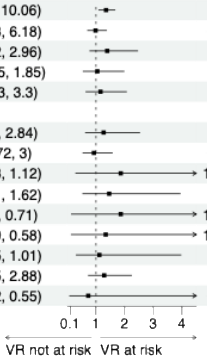

Supplementary Figure 3. Subgroup analysis of the association between virologic rebound and each post-acute COVID-19 outcome in all study participants by vaccination status for post-acute COVID-19 outcomes 21-365 days after the index date. Vaccinated cohort with virologic rebound (n = 1186) and vaccinated cohort without virologic rebound (n = 9053). Unvaccinated cohort with virologic rebound (n = 387) and unvaccinated cohort without virologic rebound (n = 3269). Adjusted HRs (square dots) and 95% (error bars) CIs are presented. The dashed vertical line represents the HR of 1.00. Statistical analysis with two-sided Wald test. VR: virologic rebound. CI: confidence interval.

(A) All patients

Outcome	Without VR Events/Number at risk	With VR Events/Number at risk	Risk difference (95% CI)	HR (95% CI)	P value
Death	1847/12322	329/1573	5.93% (3.82, 8.03)	1.69 (1.47 to 1.95)	<0.001
Composite hospitalization	1114/9327	169/1104	3.36% (1.14, 5.59)	1.25 (1.03 to 1.51)	0.022
Congestive heart failure	297/11548	43/1425	0.45% (−0.49, 1.38)	1.31 (0.92 to 1.88)	0.132
Atrial fibrillation	271/11525	44/1448	0.69% (−0.24, 1.61)	1.41 (0.98 to 2.03)	0.064
Coronary artery disease	305/11490	46/1416	0.59% (−0.37, 1.56)	1.18 (0.82 to 1.69)	0.367
Deep vein thrombosis	62/12240	2/1560	−0.38% (−0.6, −0.16)	0.45 (0.11 to 1.90)	0.274
Chronic pulmonary disease	165/11607	28/1483	0.47% (−0.26, 1.19)	1.38 (0.85 to 2.23)	0.190
Acute respiratory distress syndrome	359/12003	53/1506	0.53% (−0.45, 1.51)	1.01 (0.73 to 1.41)	0.937
Interstitial lung disease	17/12297	4/1570	0.12% (−0.14, 0.37)	1.47 (0.41 to 5.24)	0.553
Seizure	78/12080	16/1528	0.4% (−0.13, 0.93)	1.89 (1.04 to 3.43)	0.038
Anxiety	12/12277	1/1564	−0.03% (−0.17, 0.1)	0.50 (0.06 to 4.19)	0.523
Post−traumatic stress disorder	21/12241	3/1557	0.02% (−0.21, 0.25)	0.62 (0.17 to 2.35)	0.486
End−stage renal disease	38/12245	6/1563	0.07% (−0.25, 0.4)	1.52 (0.59 to 3.92)	0.382
Acute kidney injury	264/11990	37/1518	0.24% (−0.58, 1.05)	1.25 (0.84 to 1.85)	0.274
Pancreatitis	14/12279	7/1566	0.33% (0, 0.67)	4.10 (1.29 to 13.05)	0.017

0.1 1 2 3 4

VR not at risk VR at risk

(B) nirmatrelvir/ritonavir recipients

Outcome	Without VR Events/Number at risk	With VR Events/Number at risk	Risk difference (95% CI)	HR (95% CI)	P value
Death	331/3542	68/417	6.96% (3.29, 10.64)	1.72 (1.26 to 2.35)	<0.001
Composite hospitalization	237/3007	44/331	5.41% (1.63, 9.19)	1.63 (1.12 to 2.39)	0.012
Congestive heart failure	56/3420	12/396	1.39% (−0.35, 3.13)	1.81 (0.88 to 3.72)	0.107
Atrial fibrillation	58/3463	16/397	2.36% (0.37, 4.34)	2.11 (1.13 to 3.94)	0.020
Coronary artery disease	62/3410	9/391	0.48% (−1.07, 2.04)	1.00 (0.46 to 2.20)	0.994
Deep vein thrombosis	10/3528	1/415	−0.04% (−0.55, 0.46)	1.13 (0.13 to 9.68)	0.911
Chronic pulmonary disease	32/3390	6/394	0.58% (−0.67, 1.83)	1.61 (0.59 to 4.39)	0.355
Acute respiratory distress syndrome	55/3475	7/400	0.17% (−1.18, 1.52)	0.97 (0.40 to 2.36)	0.951
Interstitial lung disease	0/3540	0/417			
Seizure	14/3518	5/415	0.81% (−0.26, 1.88)	4.01 (1.18 to 13.61)	0.026
Anxiety	3/3530	0/414			
Post−traumatic stress disorder	3/3526	0/416			
End−stage renal disease	4/3532	0/416			
Acute kidney injury	54/3484	8/409	0.41% (−1, 1.81)	1.35 (0.59 to 3.08)	0.472
Pancreatitis	2/3528	2/417	0.42% (−0.24, 1.09)	7.51 (1.05 to 53.81)	0.045

0.1 1 2 3 4

VR not at risk VR at risk

(C) molnupiravir recipients

Outcome	Without VR Events/Number at risk	With VR Events/Number at risk	Risk difference (95% CI)	HR (95% CI)	P value
Death	522/3943	103/559	5.19% (1.8, 8.57)	1.77 (1.36 to 2.32)	<0.001
Composite hospitalization	315/2700	44/344	1.12% (−2.61, 4.86)	1.16 (0.81 to 1.66)	0.432
Congestive heart failure	104/3612	12/486	−0.41% (−1.89, 1.07)	1.03 (0.53 to 1.98)	0.934
Atrial fibrillation	75/3503	12/500	0.26% (−1.17, 1.68)	1.69 (0.84 to 3.39)	0.143
Coronary artery disease	99/3560	14/471	0.19% (−1.43, 1.82)	1.52 (0.80 to 2.89)	0.206
Deep vein thrombosis	13/3904	1/550	−0.15% (−0.55, 0.25)	1.42 (0.16 to 12.72)	0.756
Chronic pulmonary disease	46/3768	5/540	−0.29% (−1.18, 0.59)	0.74 (0.26 to 2.11)	0.573
Acute respiratory distress syndrome	80/3847	14/537	0.53% (−0.89, 1.95)	1.16 (0.57 to 2.37)	0.686
Interstitial lung disease	1/3937	0/559			
Seizure	28/3818	8/535	0.76% (−0.3, 1.83)	2.21 (0.92 to 5.35)	0.078
Anxiety	5/3919	1/554	0.05% (−0.32, 0.42)	1.59 (0.16 to 15.99)	0.694
Post−traumatic stress disorder	8/3906	1/550	−0.02% (−0.41, 0.36)	0.63 (0.06 to 6.31)	0.695
End−stage renal disease	16/3902	1/551	−0.23% (−0.64, 0.18)	0.56 (0.07 to 4.71)	0.591
Acute kidney injury	73/3795	13/525	0.55% (−0.85, 1.95)	1.60 (0.82 to 3.13)	0.169
Pancreatitis	6/3925	4/556	0.57% (−0.15, 1.28)	2.22 (0.37 to 13.21)	0.379

0.1 1 2 3 4

VR not at risk VR at risk

Supplementary Figure 4. Sensitivity analysis of the association between virologic rebound and each post-acute COVID-19 outcome 21-180 days after the index date. (A) all patients. Cohort with virologic rebound (n = 1573) and cohort without virologic rebound (n = 12322). (B) nirmatrelvir/ritonavir recipients. Cohort with virologic rebound (n = 417) and cohort without virologic rebound (n = 3542). (C) molnupiravir recipients. Cohort with virologic rebound (n = 559) and cohort without virologic rebound (n = 3943). Adjusted HRs (square dots) and 95% (error bars) CIs are presented. The dashed vertical line represents the HR of 1.00. Statistical analysis with two-sided Wald test. VR: virologic rebound. CI: confidence interval.

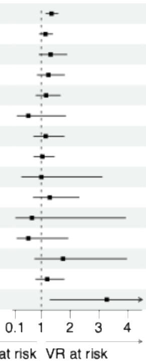

(A) All patients

Outcome	Without VR Events/Number at risk	With VR Events/Number at risk	Risk difference (95% CI)	HR (95% CI)	P value
Death	1913/10367	330/1328	6.4% (3.96, 8.84)	1.35 (1.16 to 1.58)	<0.001
Composite hospitalization	1167/7878	169/931	3.34% (0.74, 5.94)	1.14 (0.94 to 1.38)	0.171
Congestive heart failure	326/9749	47/1207	0.55% (−0.6, 1.7)	1.32 (0.93 to 1.88)	0.120
Atrial fibrillation	293/9698	42/1222	0.42% (−0.66, 1.49)	1.25 (0.86 to 1.80)	0.239
Coronary artery disease	344/9661	51/1196	0.7% (−0.5, 1.91)	1.17 (0.82 to 1.65)	0.390
Deep vein thrombosis	54/10295	3/1318	−0.3% (−0.59, 0)	0.55 (0.16 to 1.84)	0.332
Chronic pulmonary disease	194/9795	33/1255	0.65% (−0.28, 1.58)	1.15 (0.74 to 1.79)	0.527
Acute respiratory distress syndrome	336/10120	54/1271	0.93% (−0.23, 2.09)	1.04 (0.74 to 1.45)	0.825
Interstitial lung disease	16/10348	5/1325	0.22% (−0.12, 0.56)	1.00 (0.32 to 3.11)	0.996
Seizure	94/10159	16/1288	0.32% (−0.32, 0.95)	1.30 (0.73 to 2.31)	0.379
Anxiety	12/10325	2/1320	0.04% (−0.18, 0.26)	0.68 (0.12 to 3.93)	0.662
Post−traumatic stress disorder	28/10290	3/1315	−0.04% (−0.32, 0.23)	0.55 (0.16 to 1.92)	0.349
End−stage renal disease	44/10296	11/1318	0.41% (−0.1, 0.91)	1.75 (0.78 to 3.96)	0.176
Acute kidney injury	295/10089	44/1288	0.49% (−0.55, 1.54)	1.20 (0.81 to 1.79)	0.354
Pancreatitis	23/10330	8/1321	0.38% (−0.05, 0.81)	3.28 (1.31 to 8.19)	0.011

0.1 1 2 3 4
VR not at risk VR at risk

(B) nirmatrelvir/ritonavir recipients

Outcome	Without VR Events/Number at risk	With VR Events/Number at risk	Risk difference (95% CI)	HR (95% CI)	P value
Death	367/3059	86/347	12.79% (8.1, 17.47)	1.90 (1.42 to 2.53)	<0.001
Composite hospitalization	267/2609	46/274	6.55% (1.98, 11.13)	1.52 (1.05 to 2.19)	0.026
Congestive heart failure	63/2960	14/329	2.13% (−0.12, 4.37)	1.87 (0.94 to 3.70)	0.074
Atrial fibrillation	65/2988	18/330	3.28% (0.77, 5.78)	2.55 (1.40 to 4.62)	0.002
Coronary artery disease	74/2945	13/325	1.49% (−0.72, 3.69)	1.27 (0.62 to 2.57)	0.511
Deep vein thrombosis	8/3048	1/346	0.03% (−0.57, 0.62)	1.64 (0.19 to 14.10)	0.654
Chronic pulmonary disease	47/2941	7/328	0.54% (−1.09, 2.16)	1.24 (0.50 to 3.04)	0.646
Acute respiratory distress syndrome	59/3005	10/332	1.05% (−0.86, 2.95)	1.44 (0.67 to 3.10)	0.355
Interstitial lung disease	0/3059	0/347			
Seizure	16/3039	5/346	0.92% (−0.36, 2.2)	2.68 (0.85 to 8.42)	0.092
Anxiety	3/3048	0/344			
Post−traumatic stress disorder	3/3043	0/346			
End−stage renal disease	4/3049	1/346	0.16% (−0.42, 0.74)	1.74 (0.12 to 25.58)	0.686
Acute kidney injury	65/3010	10/341	0.77% (−1.09, 2.64)	1.51 (0.71 to 3.19)	0.281
Pancreatitis	4/3046	3/347	0.73% (−0.25, 1.72)	7.59 (1.50 to 38.33)	0.014

0.1 1 2 3 4
VR not at risk VR at risk

(C) molnupiravir recipients

Outcome	Without VR Events/Number at risk	With VR Events/Number at risk	Risk difference (95% CI)	HR (95% CI)	P value
Death	619/3445	103/481	3.45% (−0.44, 7.33)	1.20 (0.89 to 1.62)	0.242
Composite hospitalization	363/2370	49/297	1.18% (−3.28, 5.65)	1.26 (0.90 to 1.78)	0.181
Congestive heart failure	133/3163	14/421	−0.88% (−2.73, 0.97)	1.28 (0.69 to 2.38)	0.431
Atrial fibrillation	94/3071	12/431	−0.28% (−1.95, 1.39)	1.54 (0.79 to 3.01)	0.207
Coronary artery disease	120/3106	15/404	−0.15% (−2.11, 1.81)	1.17 (0.64 to 2.14)	0.612
Deep vein thrombosis	17/3411	2/473	−0.08% (−0.71, 0.56)	1.62 (0.28 to 9.59)	0.592
Chronic pulmonary disease	57/3297	8/466	−0.01% (−1.27, 1.25)	0.74 (0.30 to 1.83)	0.508
Acute respiratory distress syndrome	73/3367	15/460	1.09% (−0.6, 2.79)	1.49 (0.76 to 2.91)	0.243
Interstitial lung disease	2/3441	2/481	0.36% (−0.22, 0.94)	5.28 (0.49 to 57.10)	0.171
Seizure	39/3338	6/459	0.14% (−0.96, 1.24)	1.10 (0.43 to 2.84)	0.845
Anxiety	5/3422	2/477	0.27% (−0.32, 0.87)	1.49 (0.19 to 11.91)	0.707
Post−traumatic stress disorder	11/3410	1/474	−0.11% (−0.57, 0.34)	0.47 (0.05 to 4.13)	0.494
End−stage renal disease	17/3408	2/473	−0.08% (−0.71, 0.55)	0.97 (0.21 to 4.42)	0.964
Acute kidney injury	93/3315	16/456	0.7% (−1.08, 2.48)	1.33 (0.60 to 2.93)	0.482
Pancreatitis	7/3429	4/478	0.63% (−0.2, 1.46)	2.59 (0.51 to 13.10)	0.250

0.1 1 2 3 4
VR not at risk VR at risk

Supplementary Figure 5. Sensitivity analysis of the association between virologic rebound and each post-acute COVID-19 outcome 21-365 days after the index date excluding patients with virologic rebound occurred beyond 14 days post the index date. (A) all patients. Cohort with virologic rebound (n = 1328) and cohort without virologic rebound (n = 10367). (B) nirmatrelvir/ritonavir recipients. Cohort with virologic rebound (n = 347) and cohort without virologic rebound (n = 3059). (C) molnupiravir recipients. Cohort with virologic rebound (n = 481) and cohort without virologic rebound (n = 3445). Adjusted HRs (square dots) and 95% (error bars) CIs are presented. The dashed vertical line represents the HR of 1.00. Statistical analysis with two-sided Wald test. virologicVR: virologic rebound. CI: confidence interval.

(A) All patients

Outcome	Without VR Events/Number at risk	With VR Events/Number at risk	Risk difference (95% CI)	HR (95% CI)	P value
Death	2284/11578	728/2639	7.86% (6.01, 9.71)	1.30 (1.19 to 1.42)	<0.001
Composite hospitalization	1359/8842	382/1911	4.62% (2.68, 6.56)	1.13 (1.01 to 1.27)	0.041
Congestive heart failure	379/10827	103/2441	0.72% (−0.15, 1.59)	1.08 (0.86 to 1.35)	0.519
Atrial fibrillation	332/10846	105/2418	1.28% (0.41, 2.16)	1.27 (1.01 to 1.60)	0.043
Coronary artery disease	358/10788	137/2407	2.37% (1.39, 3.36)	1.45 (1.18 to 1.79)	<0.001
Deep vein thrombosis	67/11502	16/2618	0.03% (−0.3, 0.36)	1.15 (0.65 to 2.03)	0.625
Chronic pulmonary disease	223/10893	65/2483	0.57% (−0.11, 1.25)	1.08 (0.82 to 1.44)	0.576
Acute respiratory distress syndrome	391/11254	122/2567	1.28% (0.39, 2.17)	1.04 (0.84 to 1.29)	0.721
Interstitial lung disease	14/11555	10/2634	0.26% (0.02, 0.5)	1.61 (0.69 to 3.77)	0.272
Seizure	110/11358	25/2566	0.01% (−0.41, 0.43)	1.06 (0.67 to 1.66)	0.810
Anxiety	14/11537	3/2626	−0.01% (−0.15, 0.14)	0.78 (0.21 to 2.87)	0.705
Post−traumatic stress disorder	32/11500	8/2617	0.03% (−0.2, 0.26)	1.35 (0.60 to 3.04)	0.465
End−stage renal disease	61/11513	11/2611	−0.11% (−0.39, 0.17)	0.65 (0.34 to 1.27)	0.208
Acute kidney injury	323/11256	111/2564	1.46% (0.61, 2.31)	1.27 (1.01 to 1.60)	0.039
Pancreatitis	25/11537	10/2629	0.16% (−0.09, 0.41)	2.03 (0.94 to 4.37)	0.071

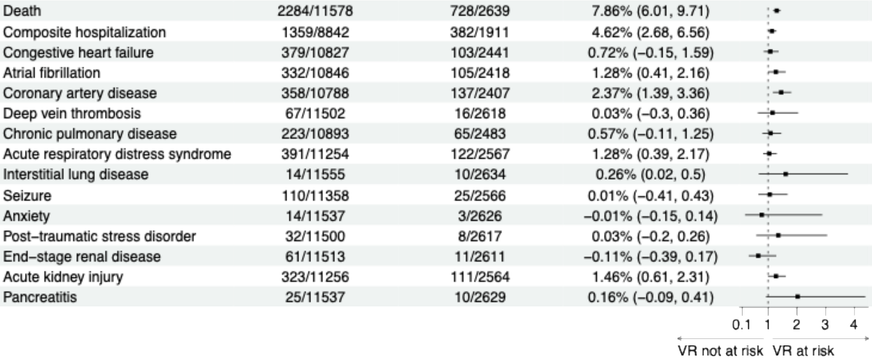

(B) nirmatrelvir/ritonavir recipients

Outcome	Without VR Events/Number at risk	With VR Events/Number at risk	Risk difference (95% CI)	HR (95% CI)	P value
Death	426/3332	177/710	12.14% (8.77, 15.52)	1.55 (1.29 to 1.87)	<0.001
Composite hospitalization	301/2841	100/580	6.65% (3.37, 9.92)	1.37 (1.08 to 1.73)	0.008
Congestive heart failure	72/3214	21/681	0.84% (−0.55, 2.24)	1.05 (0.64 to 1.74)	0.843
Atrial fibrillation	72/3265	30/673	2.25% (0.61, 3.89)	1.77 (1.14 to 2.75)	0.011
Coronary artery disease	68/3201	35/675	3.06% (1.32, 4.81)	1.96 (1.27 to 3.00)	0.002
Deep vein thrombosis	14/3317	2/708	−0.14% (−0.59, 0.31)	0.60 (0.13 to 2.71)	0.509
Chronic pulmonary disease	47/3185	17/670	1.06% (−0.2, 2.32)	1.28 (0.72 to 2.28)	0.397
Acute respiratory distress syndrome	67/3262	22/693	1.12% (−0.27, 2.51)	1.07 (0.65 to 1.76)	0.796
Interstitial lung disease	0/3330	0/710			
Seizure	22/3314	4/702	−0.09% (−0.72, 0.53)	0.91 (0.30 to 2.74)	0.869
Anxiety	4/3322	0/705			
Post−traumatic stress disorder	5/3316	0/708			
End−stage renal disease	5/3325	2/706	0.13% (−0.28, 0.55)	1.90 (0.36 to 9.96)	0.449
Acute kidney injury	69/3281	29/695	2.07% (0.5, 3.64)	1.53 (0.97 to 2.41)	0.069
Pancreatitis	5/3320	2/707	0.13% (−0.28, 0.55)	1.24 (0.24 to 6.57)	0.796

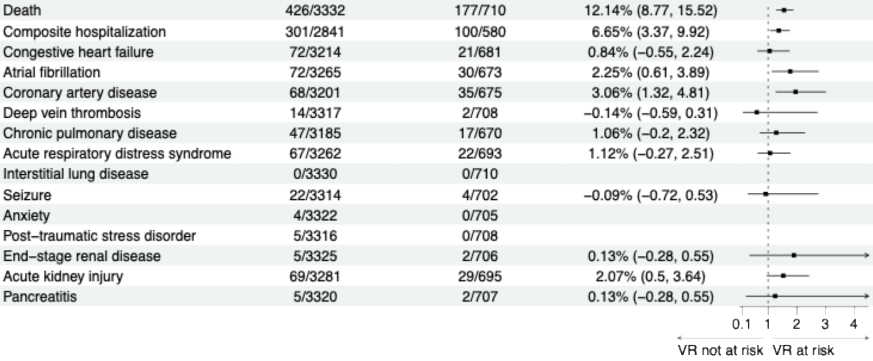

(C) molnupiravir recipients

Outcome	Without VR Events/Number at risk	With VR Events/Number at risk	Risk difference (95% CI)	HR (95% CI)	P value
Death	662/3576	266/1019	7.59% (4.61, 10.57)	1.32 (1.14 to 1.53)	<0.001
Composite hospitalization	384/2471	125/666	3.23% (−0.06, 6.52)	1.08 (0.88 to 1.34)	0.443
Congestive heart failure	131/3251	46/929	0.92% (−0.63, 2.47)	1.15 (0.82 to 1.61)	0.426
Atrial fibrillation	96/3180	38/901	1.2% (−0.24, 2.64)	1.36 (0.93 to 2.00)	0.114
Coronary artery disease	124/3216	49/902	1.58% (−0.05, 3.2)	1.33 (0.95 to 1.88)	0.096
Deep vein thrombosis	16/3538	8/1008	0.34% (−0.25, 0.93)	1.76 (0.75 to 4.14)	0.195
Chronic pulmonary disease	61/3416	16/975	−0.14% (−1.06, 0.77)	0.83 (0.48 to 1.45)	0.512
Acute respiratory distress syndrome	78/3474	37/1000	1.45% (0.19, 2.72)	1.23 (0.82 to 1.86)	0.316
Interstitial lung disease	1/3571	3/1018	0.27% (−0.07, 0.6)	5.91 (0.59 to 59.39)	0.131
Seizure	44/3469	9/974	−0.34% (−1.05, 0.36)	0.73 (0.35 to 1.52)	0.400
Anxiety	6/3553	1/1013	−0.07% (−0.31, 0.17)	0.55 (0.06 to 4.71)	0.587
Post−traumatic stress disorder	14/3544	3/1004	−0.1% (−0.49, 0.3)	0.85 (0.24 to 3.08)	0.806
End−stage renal disease	24/3540	2/1004	−0.48% (−0.86, −0.09)	0.27 (0.06 to 1.14)	0.074
Acute kidney injury	91/3430	40/979	1.43% (0.08, 2.78)	1.30 (0.88 to 1.92)	0.187
Pancreatitis	6/3558	6/1016	0.42% (−0.07, 0.91)	3.75 (1.17 to 11.96)	0.026

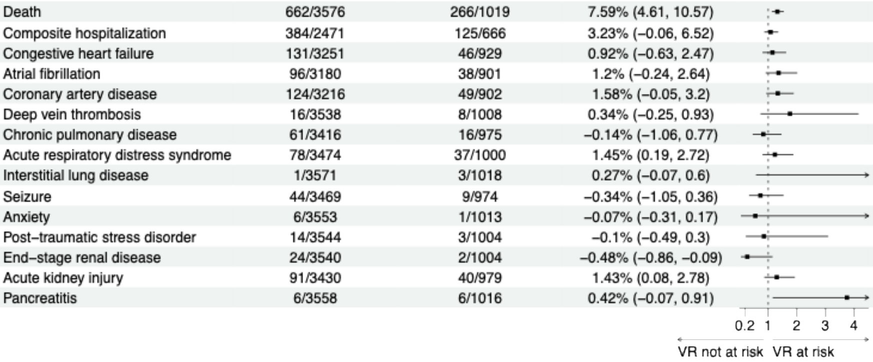

Supplementary Figure 6. Sensitivity analysis of the association between virologic rebound and each post-acute COVID-19 outcome 21-365 days after the index date, using alternative definition of virologic rebound (i): a decrease in Ct value of at least 3 units after the end of oral antiviral treatment or treatment completion proxy within 21 days post the index date of virologic rebound. (A) all patients. Cohort with virologic rebound (n = 2639) and cohort without virologic rebound (n = 11578). (B) nirmatrelvir/ritonavir recipients. Cohort with virologic rebound (n = 710) and cohort without virologic rebound (n = 3332). (C) molnupiravir recipients. Cohort with virologic rebound (n = 1019) and cohort without virologic rebound (n = 3576). virologicvirologicAdjusted HRs (square dots) and 95% (error bars) CIs are presented. The dashed vertical line represents the HR of 1.00. Statistical analysis with two-sided Wald test. VR: virologic rebound. CI: confidence interval.

(A) All patients

Outcome	Without VR Events/Number at risk	With VR Events/Number at risk	Risk difference (95% CI)	HR (95% CI)	P value
Death	2350/11399	545/2228	3.85% (1.91, 5.78)	1.45 (1.29 to 1.62)	<0.001
Composite hospitalization	1313/8658	303/1631	3.41% (1.38, 5.45)	1.25 (1.05 to 1.49)	0.012
Congestive heart failure	384/10701	74/2041	0.04% (−0.85, 0.92)	1.14 (0.86 to 1.53)	0.359
Atrial fibrillation	328/10668	79/2065	0.75% (−0.14, 1.64)	1.35 (1.01 to 1.80)	0.042
Coronary artery disease	371/10622	97/2042	1.26% (0.27, 2.24)	1.43 (1.09 to 1.86)	0.009
Deep vein thrombosis	66/11318	13/2212	0% (−0.34, 0.35)	2.08 (1.03 to 4.18)	0.041
Chronic pulmonary disease	212/10731	48/2102	0.31% (−0.38, 1)	1.28 (0.88 to 1.87)	0.204
Acute respiratory distress syndrome	396/11097	93/2153	0.75% (−0.17, 1.68)	1.15 (0.87 to 1.51)	0.317
Interstitial lung disease	15/11374	8/2226	0.23% (−0.03, 0.48)	2.79 (0.80 to 9.70)	0.106
Seizure	105/11178	23/2172	0.12% (−0.35, 0.59)	1.29 (0.78 to 2.13)	0.326
Anxiety	10/11360	6/2215	0.18% (−0.04, 0.41)	3.51 (1.07 to 11.52)	0.038
Post−traumatic stress disorder	27/11317	8/2210	0.12% (−0.14, 0.39)	1.11 (0.43 to 2.87)	0.833
End−stage renal disease	55/11322	13/2216	0.1% (−0.24, 0.44)	1.31 (0.67 to 2.57)	0.428
Acute kidney injury	318/11098	82/2155	0.94% (0.07, 1.81)	1.45 (1.07 to 1.97)	0.016
Pancreatitis	23/11359	7/2221	0.11% (−0.13, 0.36)	1.60 (0.60 to 4.24)	0.343

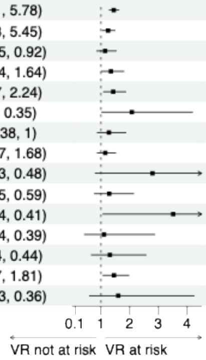

(B) nirmatrelvir/ritonavir recipients

Outcome	Without VR Events/Number at risk	With VR Events/Number at risk	Risk difference (95% CI)	HR (95% CI)	P value
Death	440/3207	139/666	7.15% (3.84, 10.46)	1.59 (1.23 to 2.04)	<0.001
Composite hospitalization	285/2731	92/546	6.41% (3.07, 9.76)	1.59 (1.16 to 2.19)	0.004
Congestive heart failure	70/3096	22/638	1.19% (−0.32, 2.7)	1.59 (0.87 to 2.89)	0.131
Atrial fibrillation	72/3133	27/641	1.91% (0.27, 3.56)	1.61 (0.96 to 2.71)	0.069
Coronary artery disease	72/3085	24/634	1.45% (−0.13, 3.03)	1.32 (0.72 to 2.44)	0.366
Deep vein thrombosis	10/3194	6/662	0.59% (−0.15, 1.34)	11.32 (3.40 to 37.68)	<0.001
Chronic pulmonary disease	48/3063	9/635	−0.15% (−1.17, 0.87)	0.60 (0.25 to 1.46)	0.261
Acute respiratory distress syndrome	69/3150	14/640	0% (−1.25, 1.24)	1.12 (0.58 to 2.16)	0.742
Interstitial lung disease	0/3205	0/666			
Seizure	17/3186	8/663	0.67% (−0.2, 1.54)	3.27 (1.24 to 8.62)	0.016
Anxiety	3/3198	1/661	0.06% (−0.26, 0.37)	2.82 (0.28 to 28.61)	0.382
Post−traumatic stress disorder	3/3193	1/662	0.06% (−0.26, 0.37)	2.40 (0.21 to 26.92)	0.478
End−stage renal disease	4/3197	3/665	0.33% (−0.2, 0.85)	5.58 (0.76 to 41.23)	0.092
Acute kidney injury	71/3159	22/653	1.12% (−0.36, 2.6)	1.57 (0.86 to 2.87)	0.140
Pancreatitis	3/3194	3/666	0.36% (−0.16, 0.88)	5.40 (0.89 to 32.64)	0.066

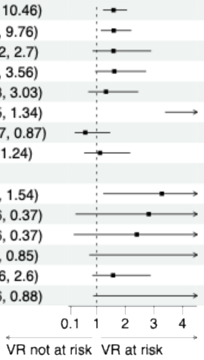

(C) molnupiravir recipients

Outcome	Without VR Events/Number at risk	With VR Events/Number at risk	Risk difference (95% CI)	HR (95% CI)	P value
Death	697/3597	194/798	4.93% (1.69, 8.18)	1.51 (1.22 to 1.86)	<0.001
Composite hospitalization	387/2486	85/509	1.13% (−2.41, 4.67)	1.12 (0.85 to 1.48)	0.410
Congestive heart failure	146/3315	23/697	−1.1% (−2.6, 0.39)	0.86 (0.53 to 1.42)	0.562
Atrial fibrillation	92/3208	29/707	1.23% (−0.34, 2.81)	1.82 (1.11 to 2.98)	0.018
Coronary artery disease	132/3241	33/697	0.66% (−1.06, 2.38)	1.62 (1.04 to 2.51)	0.032
Deep vein thrombosis	21/3560	3/786	−0.21% (−0.71, 0.29)	1.87 (0.51 to 6.89)	0.348
Chronic pulmonary disease	55/3441	14/766	0.23% (−0.81, 1.27)	1.28 (0.62 to 2.62)	0.506
Acute respiratory distress syndrome	85/3506	27/774	1.06% (−0.33, 2.45)	1.08 (0.60 to 1.92)	0.803
Interstitial lung disease	1/3591	3/798	0.35% (−0.08, 0.78)	3.43 (0.34 to 34.52)	0.296
Seizure	42/3488	10/765	0.1% (−0.78, 0.99)	1.12 (0.53 to 2.38)	0.763
Anxiety	4/3576	2/791	0.14% (−0.23, 0.51)	1.83 (0.30 to 11.21)	0.512
Post−traumatic stress disorder	11/3558	3/790	0.07% (−0.4, 0.54)	0.92 (0.17 to 4.85)	0.917
End−stage renal disease	24/3557	2/788	−0.42% (−0.86, 0.02)	0.40 (0.09 to 1.73)	0.219
Acute kidney injury	95/3461	24/758	0.42% (−0.94, 1.78)	1.06 (0.60 to 1.87)	0.841
Pancreatitis	8/3581	4/794	0.28% (−0.24, 0.8)	1.71 (0.40 to 7.41)	0.470

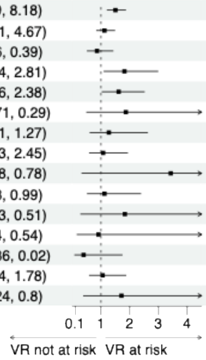

Supplementary Figure 7. Sensitivity analysis of the association between virologic rebound and each post-acute COVID-19 outcome 21-365 days after the index date, using alternative definition of virologic rebound (ii): at least two consecutive Ct measurements with values larger than or equal to 30 followed by at least two consecutive values less than 30. (A) all patients. Cohort with virologic rebound (n = 2228) and cohort without virologic rebound (n = 11399). (B) nirmatrelvir/ritonavir recipients. Cohort with virologic rebound (n = 666) and cohort without virologic rebound (n = 3207). (C) molnupiravir recipients. Cohort with virologic rebound (n = 798) and cohort without virologic rebound (n = 3597). virologicAdjusted HRs (square dots) and 95% (error bars) CIs are presented. The dashed vertical line represents the HR of 1.00. Statistical analysis with two-sided Wald test. VR: virologic rebound. CI: confidence interval.

(A) All patients

Outcome	Without VR Events/Number at risk	With VR Events/Number at risk	Risk difference (95% CI)	HR (95% CI)	P value
Death	2437/11991	316/1342	3.22% (0.84, 5.6)	1.53 (1.33 to 1.76)	<0.001
Composite hospitalization	1376/9098	171/1005	1.89% (−0.55, 4.33)	1.39 (1.16 to 1.66)	<0.001
Congestive heart failure	379/11232	54/1237	0.99% (−0.2, 2.18)	1.62 (1.15 to 2.27)	0.006
Atrial fibrillation	345/11209	38/1261	−0.06% (−1.06, 0.93)	1.19 (0.82 to 1.74)	0.364
Coronary artery disease	407/11151	43/1247	−0.2% (−1.27, 0.87)	1.13 (0.80 to 1.60)	0.490
Deep vein thrombosis	59/11912	13/1331	0.48% (−0.06, 1.02)	3.88 (1.93 to 7.80)	<0.001
Chronic pulmonary disease	226/11307	26/1266	0.05% (−0.77, 0.88)	1.15 (0.72 to 1.83)	0.568
Acute respiratory distress syndrome	424/11688	46/1294	−0.07% (−1.14, 0.99)	1.27 (0.91 to 1.77)	0.161
Interstitial lung disease	18/11966	5/1339	0.22% (−0.11, 0.56)	4.39 (1.39 to 13.82)	0.011
Seizure	109/11754	13/1316	0.06% (−0.5, 0.62)	1.31 (0.70 to 2.44)	0.395
Anxiety	14/11948	3/1335	0.11% (−0.15, 0.37)	1.93 (0.46 to 8.16)	0.369
Post−traumatic stress disorder	29/11909	4/1328	0.06% (−0.25, 0.37)	0.64 (0.20 to 2.06)	0.451
End−stage renal disease	50/11915	11/1333	0.41% (−0.09, 0.9)	1.90 (0.80 to 4.51)	0.144
Acute kidney injury	334/11660	42/1307	0.35% (−0.65, 1.35)	1.33 (0.90 to 1.95)	0.148
Pancreatitis	28/11950	5/1335	0.14% (−0.2, 0.48)	1.63 (0.55 to 4.82)	0.376

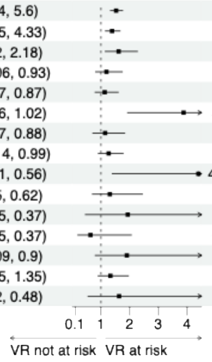

(B) nirmatrelvir/ritonavir recipients

Outcome	Without VR Events/Number at risk	With VR Events/Number at risk	Risk difference (95% CI)	HR (95% CI)	P value
Death	477/3479	70/348	6.4% (2.04, 10.77)	1.89 (1.43 to 2.50)	<0.001
Composite hospitalization	329/2965	34/279	1.09% (−2.91, 5.09)	1.34 (0.91 to 1.97)	0.145
Congestive heart failure	75/3364	14/328	2.04% (−0.2, 4.28)	2.51 (1.34 to 4.72)	0.004
Atrial fibrillation	79/3401	12/334	1.27% (−0.79, 3.33)	1.83 (0.95 to 3.52)	0.071
Coronary artery disease	90/3339	6/335	−0.9% (−2.43, 0.62)	0.83 (0.35 to 1.94)	0.660
Deep vein thrombosis	11/3465	4/346	0.84% (−0.3, 1.98)	10.03 (2.90 to 34.69)	<0.001
Chronic pulmonary disease	55/3326	2/329	−1.05% (−1.99, −0.1)	0.34 (0.08 to 1.49)	0.153
Acute respiratory distress syndrome	77/3417	6/335	−0.46% (−1.97, 1.04)	1.04 (0.44 to 2.46)	0.935
Interstitial lung disease	0/3477	0/348			
Seizure	19/3459	4/344	0.61% (−0.55, 1.77)	2.29 (0.72 to 7.23)	0.159
Anxiety	3/3467	1/346	0.2% (−0.37, 0.78)	5.05 (0.48 to 52.59)	0.176
Post−traumatic stress disorder	3/3463	1/346	0.2% (−0.37, 0.78)	1.37 (0.12 to 15.59)	0.798
End−stage renal disease	6/3469	1/347	0.12% (−0.47, 0.7)	2.79 (0.25 to 31.20)	0.405
Acute kidney injury	76/3426	8/339	0.14% (−1.55, 1.83)	1.41 (0.64 to 3.09)	0.389
Pancreatitis	5/3465	1/347	0.14% (−0.43, 0.72)	4.83 (0.53 to 43.74)	0.162

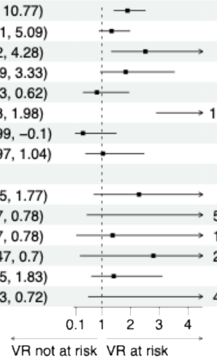

(C) molnupiravir recipients

Outcome	Without VR Events/Number at risk	With VR Events/Number at risk	Risk difference (95% CI)	HR (95% CI)	P value
Death	776/3980	76/344	2.6% (−1.96, 7.15)	1.69 (1.28 to 2.23)	<0.001
Composite hospitalization	417/2730	37/229	0.88% (−4.07, 5.84)	1.41 (0.97 to 2.03)	0.069
Congestive heart failure	149/3643	11/304	−0.47% (−2.67, 1.72)	1.26 (0.65 to 2.45)	0.501
Atrial fibrillation	111/3540	7/314	−0.91% (−2.64, 0.82)	1.18 (0.52 to 2.67)	0.698
Coronary artery disease	144/3575	15/301	0.96% (−1.59, 3.5)	2.02 (1.11 to 3.66)	0.021
Deep vein thrombosis	21/3942	2/338	0.06% (−0.79, 0.91)	5.85 (1.28 to 26.69)	0.023
Chronic pulmonary disease	60/3812	8/332	0.84% (−0.86, 2.53)	1.86 (0.72 to 4.78)	0.199
Acute respiratory distress syndrome	97/3881	11/335	0.78% (−1.19, 2.75)	1.65 (0.82 to 3.35)	0.163
Interstitial lung disease	4/3975	0/343			
Seizure	47/3854	4/335	−0.03% (−1.24, 1.19)	0.95 (0.32 to 2.79)	0.919
Anxiety	6/3957	1/340	0.14% (−0.45, 0.73)	3.09 (0.33 to 28.76)	0.321
Post−traumatic stress disorder	11/3939	2/340	0.31% (−0.52, 1.14)	2.13 (0.41 to 11.02)	0.366
End−stage renal disease	19/3941	3/337	0.41% (−0.62, 1.43)	2.73 (0.76 to 9.86)	0.125
Acute kidney injury	106/3821	9/330	−0.05% (−1.88, 1.79)	1.21 (0.53 to 2.72)	0.651
Pancreatitis	10/3963	2/342	0.33% (−0.49, 1.16)	2.20 (0.35 to 13.96)	0.402

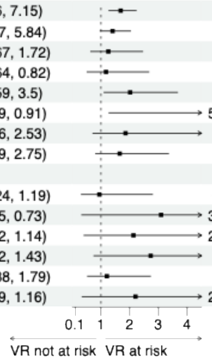

Supplementary Figure 8. Sensitivity analysis of the association between virologic rebound and each post-acute COVID-19 outcome 21-365 days after the index date, using alternative definition of virologic rebound (iii): a reduction in two consecutive Ct values from a value larger than 40 to a value less than or equal to 40. (A) all patients. Cohort with virologic rebound (n = 1342) and cohort without virologic rebound (n = 11991). (B) nirmatrelvir/ritonavir recipients. Cohort with virologic rebound (n = 348) and cohort without virologic rebound (n = 3479). (C) molnupiravir recipients. Cohort with virologic rebound (n = 344) and cohort without virologic rebound (n = 3980). virologicAdjusted HRs (square dots) and 95% (error bars) CIs are presented. The dashed vertical line represents the HR of 1.00. Statistical analysis with two-sided Wald test. VR: virologic rebound. CI: confidence interval.

(A) All patients

Outcome	Without VR Events/Number at risk	With VR Events/Number at risk	Risk difference (95% CI)	HR (95% CI)	P value
Death	2272/11416	321/1185	7.19% (4.55, 9.82)	1.46 (1.29 to 1.65)	<0.001
Composite hospitalization	1346/8633	165/840	4.05% (1.26, 6.85)	1.19 (1.00 to 1.41)	0.053
Congestive heart failure	384/10707	47/1080	0.77% (−0.5, 2.03)	1.37 (1.00 to 1.88)	0.051
Atrial fibrillation	338/10675	42/1093	0.68% (−0.51, 1.86)	1.26 (0.90 to 1.77)	0.180
Coronary artery disease	397/10636	51/1060	1.08% (−0.26, 2.42)	1.22 (0.89 to 1.67)	0.210
Deep vein thrombosis	66/11340	3/1175	−0.33% (−0.65, −0.01)	0.60 (0.19 to 1.91)	0.388
Chronic pulmonary disease	227/10764	34/1121	0.92% (−0.12, 1.96)	1.33 (0.90 to 1.96)	0.157
Acute respiratory distress syndrome	399/11124	54/1137	1.16% (−0.12, 2.45)	1.02 (0.76 to 1.38)	0.888
Interstitial lung disease	15/11394	5/1183	0.29% (−0.08, 0.67)	1.96 (0.65 to 5.96)	0.233
Seizure	103/11187	15/1151	0.38% (−0.3, 1.06)	1.50 (0.86 to 2.64)	0.156
Anxiety	13/11373	1/1183	−0.03% (−0.21, 0.15)	0.61 (0.08 to 4.94)	0.644
Post−traumatic stress disorder	31/11344	2/1174	−0.1% (−0.36, 0.15)	0.39 (0.09 to 1.78)	0.226
End−stage renal disease	56/11345	8/1178	0.19% (−0.3, 0.67)	1.14 (0.51 to 2.54)	0.753
Acute kidney injury	330/11107	37/1146	0.26% (−0.81, 1.33)	1.11 (0.78 to 1.60)	0.554
Pancreatitis	23/11376	6/1181	0.31% (−0.11, 0.72)	3.15 (1.20 to 8.22)	0.019

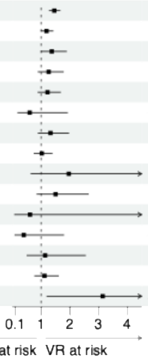

(B) nirmatrelvir/ritonavir recipients

Outcome	Without VR Events/Number at risk	With VR Events/Number at risk	Risk difference (95% CI)	HR (95% CI)	P value
Death	442/3346	80/324	11.48% (6.65, 16.32)	1.65 (1.27 to 2.13)	<0.001
Composite hospitalization	311/2839	47/263	6.92% (2.15, 11.69)	1.50 (1.07 to 2.09)	0.018
Congestive heart failure	75/3230	14/311	2.18% (−0.18, 4.54)	1.78 (0.94 to 3.34)	0.075
Atrial fibrillation	75/3271	16/308	2.9% (0.37, 5.43)	1.79 (1.00 to 3.20)	0.051
Coronary artery disease	81/3218	13/302	1.79% (−0.56, 4.14)	1.37 (0.72 to 2.61)	0.340
Deep vein thrombosis	11/3334	1/323	−0.02% (−0.66, 0.62)	0.85 (0.10 to 6.96)	0.879
Chronic pulmonary disease	55/3205	8/307	0.89% (−0.95, 2.73)	1.21 (0.55 to 2.66)	0.629
Acute respiratory distress syndrome	71/3282	9/313	0.71% (−1.21, 2.63)	1.07 (0.52 to 2.20)	0.856
Interstitial lung disease	0/3344	0/324			
Seizure	19/3325	5/323	0.98% (−0.39, 2.35)	2.54 (0.89 to 7.29)	0.083
Anxiety	3/3334	0/323			
Post−traumatic stress disorder	4/3331	0/323			
End−stage renal disease	6/3337	0/324			
Acute kidney injury	75/3292	10/320	0.85% (−1.13, 2.82)	1.44 (0.72 to 2.87)	0.306
Pancreatitis	4/3333	2/324	0.5% (−0.36, 1.36)	6.07 (1.04 to 35.33)	0.045

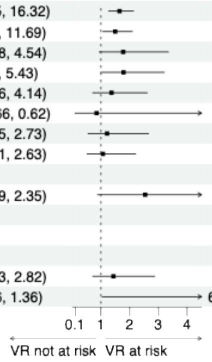

(C) molnupiravir recipients

Outcome	Without VR Events/Number at risk	With VR Events/Number at risk	Risk difference (95% CI)	HR (95% CI)	P value
Death	701/3697	96/403	4.86% (0.51, 9.21)	1.41 (1.11 to 1.79)	0.005
Composite hospitalization	411/2524	43/249	0.99% (−3.93, 5.9)	1.12 (0.80 to 1.56)	0.513
Congestive heart failure	149/3393	14/352	−0.41% (−2.57, 1.74)	1.23 (0.70 to 2.17)	0.478
Atrial fibrillation	108/3283	12/362	0.03% (−1.92, 1.97)	1.48 (0.79 to 2.78)	0.222
Coronary artery disease	142/3334	15/338	0.18% (−2.12, 2.48)	1.23 (0.70 to 2.16)	0.474
Deep vein thrombosis	21/3659	2/396	−0.07% (−0.81, 0.67)	1.60 (0.37 to 6.99)	0.531
Chronic pulmonary disease	62/3532	5/391	−0.48% (−1.67, 0.72)	0.67 (0.26 to 1.77)	0.423
Acute respiratory distress syndrome	91/3604	14/386	1.1% (−0.83, 3.04)	1.25 (0.67 to 2.32)	0.478
Interstitial lung disease	2/3691	2/403	0.44% (−0.25, 1.13)	8.10 (0.83 to 79.19)	0.072
Seizure	44/3577	5/382	0.08% (−1.12, 1.27)	1.20 (0.46 to 3.14)	0.706
Anxiety	5/3674	1/403	0.11% (−0.39, 0.61)	1.38 (0.14 to 13.17)	0.780
Post−traumatic stress disorder	14/3663	0/396			
End−stage renal disease	24/3658	1/397	−0.4% (−0.96, 0.15)	0.42 (0.05 to 3.27)	0.406
Acute kidney injury	103/3557	13/377	0.55% (−1.37, 2.48)	1.08 (0.57 to 2.03)	0.814
Pancreatitis	7/3680	3/402	0.56% (−0.3, 1.41)	4.97 (0.87 to 28.26)	0.070

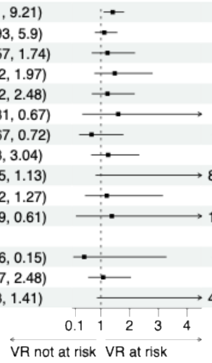

Supplementary Figure 9. Sensitivity analysis of the association between virologic rebound and each post-acute COVID-19 outcome 21-365 days after the index date in patients who were admitted at the time or after the initial positive RT-PCR. (A) all patients. Cohort with virologic rebound (n = 1185) and cohort without virologic rebound (n = 11416). (B) nirmatrelvir/ritonavir recipients. Cohort with virologic rebound (n = 324) and cohort without virologic rebound (n = 3346). (C) molnupiravir recipients. Cohort with virologic rebound (n = 403) and cohort without virologic rebound (n = 3697). Adjusted HRs (square dots) and 95% (error bars) CIs are presented. The dashed vertical line represents the HR of 1.00. Statistical analysis with two-sided Wald test. VR: virologic rebound. CI: confidence interval.

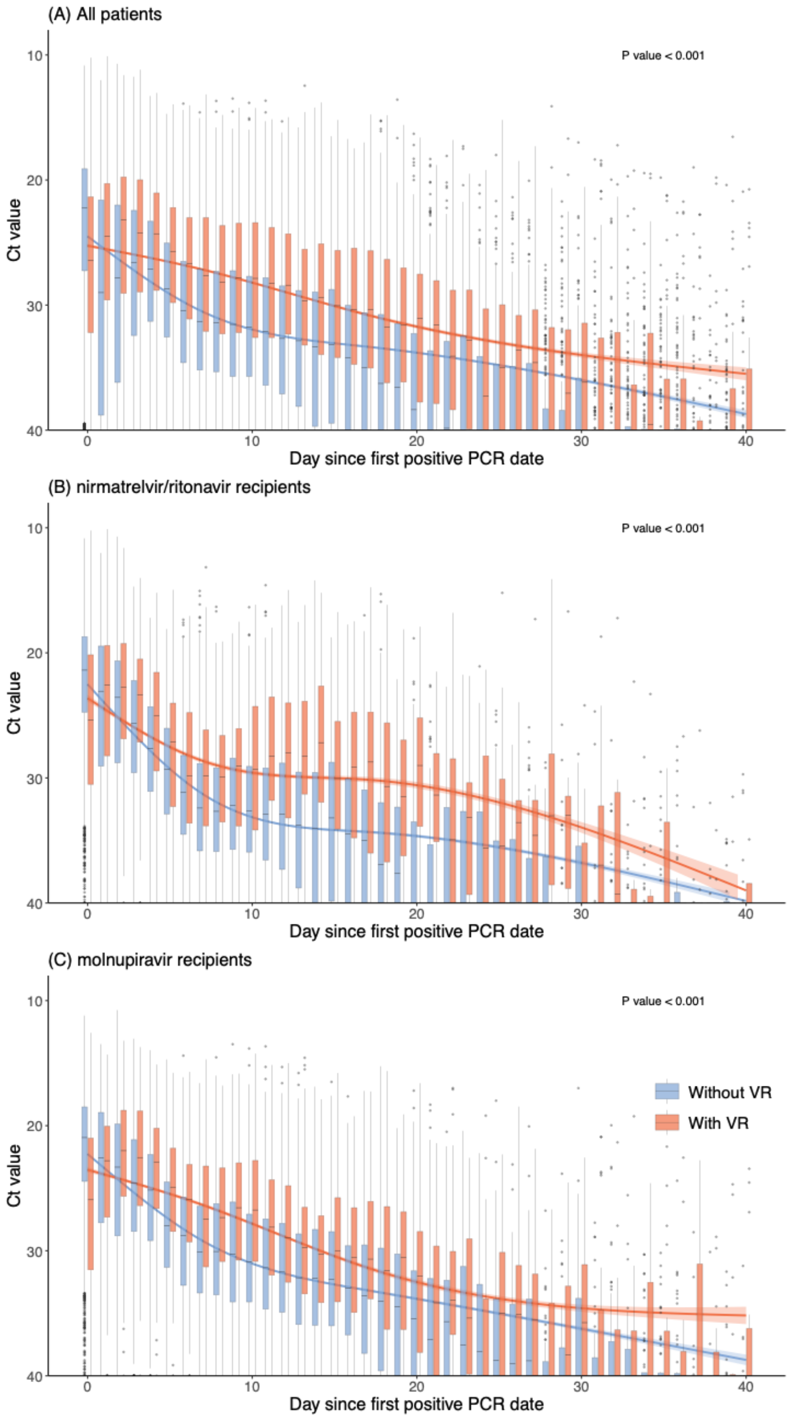

Supplementary Figure 10. Daily Ct value in (A) all patients, (B) nirmatrelvir/ritonavir recipients, and (C) molnupiravir recipients with (red) and without virologic rebound (blue) within 40 days after the index date. To compare the difference in Ct values between patients with and without virologic rebound, p-values of the effect of virologic rebound on Ct values were obtained using a generalized-additive mixed-effect model with random effect of patient-level intercept and fixed effects of covariates. Trend of Ct values throughout the 40 days post the index date was estimated by natural cubic spline with the knots set at 7 days and 17 days to aid in visualizing the pattern. The lines are predicted mean daily Ct values from the generalized-additive mixed-effect model. Statistical analysis with two-sided Wald test. Ct: cycle threshold. PCR: polymerase chain reaction.

References

1. Knol MJ, VanderWeele TJ, Groenwold RHH, Klungel OH, Rovers MM, Grobbee DE. Estimating measures of interaction on an additive scale for preventive exposures. Eur J Epidemiol. 2011;26(6):433–8.
2. Li R, Chambless L. Test for additive interaction in proportional hazards models. Ann Epidemiol. 2007;17(3):227–36.
3. World Health Organization, Manual of the International Classification of Diseases, Injuries, and Causes of Death, Ninth Revision. Geneva: World Health Organization, 1977. [cited 2025 Mar 13]. Available from: https://web.archive.org/web/20191228233120/https://simba.isr.umich.edu/restricted/docs/Mortality/icd_09_codes.pdf
