# Supplementary material for: SARS-CoV-2 rebound and post-acute mortality and hospitalization among patients admitted with COVID-19: cohort study
Source: Nat Commun. 2025 Jul 28;16:6924. doi: 10.1038/s41467-025-61737-7 (PMC12304394; doi:10.1038/s41467-025-61737-7)
Supplement: Supplementary file 2 — Reporting Summary [file 41467_2025_61737_MOESM2_ESM.pdf]

nature portfolio

Corresponding author(s): Eng Kiong Yeoh and Zihao Guo; DAPR; NCOMMS-24-55990-T

Last updated by author(s): Jun 11, 2025

Reporting Summary

Nature Portfolio wishes to improve the reproducibility of the work that we publish. This form provides structure for consistency and transparency in reporting. For further information on Nature Portfolio policies, see our Editorial Policies and the Editorial Policy Checklist.

Statistics

For all statistical analyses, confirm that the following items are present in the figure legend, table legend, main text, or Methods section.

n/a	Confirmed	
☐	☒	The exact sample size (*n*) for each experimental group/condition, given as a discrete number and unit of measurement
☒	☐	A statement on whether measurements were taken from distinct samples or whether the same sample was measured repeatedly
☒	☐	The statistical test(s) used AND whether they are one- or two-sided<br>*Only common tests should be described solely by name; describe more complex techniques in the Methods section.*
☐	☒	A description of all covariates tested
☐	☒	A description of any assumptions or corrections, such as tests of normality and adjustment for multiple comparisons
☐	☒	A full description of the statistical parameters including central tendency (e.g. means) or other basic estimates (e.g. regression coefficient) AND variation (e.g. standard deviation) or associated estimates of uncertainty (e.g. confidence intervals)
☒	☐	For null hypothesis testing, the test statistic (e.g. *F*, *t*, *r*) with confidence intervals, effect sizes, degrees of freedom and *P* value noted<br>*Give P values as exact values whenever suitable.*
☒	☐	For Bayesian analysis, information on the choice of priors and Markov chain Monte Carlo settings
☒	☐	For hierarchical and complex designs, identification of the appropriate level for tests and full reporting of outcomes
☐	☒	Estimates of effect sizes (e.g. Cohen's *d*, Pearson's *r*), indicating how they were calculated

Our web collection on statistics for biologists contains articles on many of the points above.

Software and code

Policy information about availability of computer code

Data collection	Data collection was performed in Excel and R 4.2.2, with dplyr (v.1.1.4) package.
Data analysis	Data analysis was performed in R 4.2.2, with dplyr (v.1.1.4), WeightIt (v.0.14.2), survival (v.3.7-0), ggplot2 (3.5.1) packages.

For manuscripts utilizing custom algorithms or software that are central to the research but not yet described in published literature, software must be made available to editors and reviewers. We strongly encourage code deposition in a community repository (e.g. GitHub). See the Nature Portfolio guidelines for submitting code & software for further information.

Data

Policy information about availability of data

All manuscripts must include a data availability statement. This statement should provide the following information, where applicable:

- Accession codes, unique identifiers, or web links for publicly available datasets
- A description of any restrictions on data availability
- For clinical datasets or third party data, please ensure that the statement adheres to our policy

The Hong Kong Hospital Authority and Department of Health, The Government of the Hong Kong Special Administrative Region, are the data custodians, and data requests to these parties can be made via email (hacpaaedr@ha.org.hk) and website (https://www.dh.gov.hk/english/aboutus/aboutus_pps/aboutus_pps.html), respectively. The cases' surveillance data and medication records were extracted from electronic records in the system managed by the Hong Kong Hospital Authority. The vaccine history was extracted from the COVID-19 surveillance database provided by the Department of Health in Hong Kong. Restrictions apply to the availability of these data, which were used under an agreement for the purposes of scientific research. The authors do not have the right to transfer or release the

data, in whole or in part, and in whatever form or media, or to any other parties or place outside of Hong Kong, and must fully comply with the duties under the law relating to the protection of personal data, including those under the Personal Data (Privacy) Ordinance and its principles in all aspects.

Research involving human participants, their data, or biological material

Policy information about studies with human participants or human data. See also policy information about sex, gender (identity/presentation), and sexual orientation and race, ethnicity and racism.

Reporting on sex and gender	The sex of the study participants was self-reported. Table 1 of the manuscript provides summary statistics on sex information.
Reporting on race, ethnicity, or other socially relevant groupings	No information on race, ethnicity, or other socially relevant groupings was reported in the study, and this information was not available in the database.
Population characteristics	The baseline characteristics of the study participants including age, sex, comorbidities, COVID-19 vaccination status, prescription of pharmaceutical and non-pharmaceutical interventions, and cycle threshold values were reported in Table 1 of the manuscript.
Recruitment	Population-based electronic health database provided by the Hospital Authority of Hong Kong.
Ethics oversight	Ethics approval was obtained from the Joint CUHK-NTEC Clinical Research Ethics Committee (No. 2023.006).

Note that full information on the approval of the study protocol must also be provided in the manuscript.

Field-specific reporting

Please select the one below that is the best fit for your research. If you are not sure, read the appropriate sections before making your selection.

[x] Life sciences [ ] Behavioural & social sciences [ ] Ecological, evolutionary & environmental sciences

For a reference copy of the document with all sections, see nature.com/documents/nr-reporting-summary-flat.pdf

Life sciences study design

All studies must disclose on these points even when the disclosure is negative.

Sample size	A total of 88,643 hospitalized patients with confirmed SARS-CoV-2 infection from March 11, 2022 to October 10, 2023 were identified in Hong Kong were identified. Collectively 26,517 (1,758 with virological rebound, 24,759 without virological rebound) patients were eligible for inclusion for analysis, with 6,708 (468 with virological rebound, 6,240 without virological rebound) of them received Nirmatrelvir/ritonavir and 7,710 of them received Molnupiravir (616 with virological rebound, 7,710 without virological rebound) as oral antiviral treatment against COVID-19.
Data exclusions	We excluded hospitalized patients with confirmed SARS-CoV-2 infection who (1) were aged < 18 years; (2) died within 21 days after the first test-positive date of SARS-CoV-2; (3) did not meet the criteria of antiviral treatment group or non-antiviral treatment group; (4) had no cycle threshold measurements available; (5) received oral antiviral treatment without at least one cycle threshold value before or during the antiviral treatment, and without at least one cycle threshold value after the end of antiviral treatment; (6) did not receive antiviral treatment and did not have at least one cycle threshold value measurements 5 days after the initial Ct value measurement; or (7) had virological rebound occurred beyond 21 days post the first test-positive date.
Replication	Interest parties could liaise the Hospital Authority and the Center for Health and Protection of Hong Kong for acquisition of de-identified clinical data and vaccination record.<br><br>Please note that the above data is necessary for reproducing our results.
Randomization	Randomization is not relevant to our study because this is a retrospective cohort study.
Blinding	Blinding is not possible because this is a retrospective cohort study.

Reporting for specific materials, systems and methods

We require information from authors about some types of materials, experimental systems and methods used in many studies. Here, indicate whether each material, system or method listed is relevant to your study. If you are not sure if a list item applies to your research, read the appropriate section before selecting a response.

Materials & experimental systems

n/a	Involved in the study
☒	☐ Antibodies
☒	☐ Eukaryotic cell lines
☒	☐ Palaeontology and archaeology
☒	☐ Animals and other organisms
☒	☐ Clinical data
☒	☐ Dual use research of concern
☒	☐ Plants

Methods

n/a	Involved in the study
☒	☐ ChIP-seq
☒	☐ Flow cytometry
☒	☐ MRI-based neuroimaging

Plants

Seed stocks	n/a
Novel plant genotypes	n/a
Authentication	n/a
